# Effect of olanzapine combined with risperidone in the treatment of schizophrenia and its influence on cognitive function

**DOI:** 10.12669/pjms.37.3.3348

**Published:** 2021

**Authors:** Lujie Yang, Xia Qi

**Affiliations:** 1 Lujie Yang, Department of Pharmacy, Binzhou Youfu Hospital, Shandong, 256613, China; 2 Xia Qi, Psychiatric Women’s Ward, Binzhou Youfu Hospital, Shandong, 256613, China

**Keywords:** Clinical efficacy, Cognitive function, Schizophrenia, Olanzapine, Risperidone

## Abstract

**Objective:**

To explore the effect of risperidone combined with olanzapine in the treatment of schizophrenia and its influence on cognitive function.

**Methods:**

Ninety-eight schizophrenic patients in our hospital who were admitted and treated between June 2018 and December 2019 were selected. The study group was treated with risperidone combined with olanzapine, and the control group was treated with risperidone. The clinical efficacy, Positive and Negative Syndrome Scale (PANSS) score, Wisconsin Card Sorting Test (WCST) result and adverse reactions of the two groups were compared.

**Results:**

After treatment, the scores of classification completion number and correct times of the observation group were higher than those of the control group, and the scores of the random error number and continuous error number were higher than those of the control group; the differences were statistically significant (P<0.05). The total effective rate of the observation group was 95.56%, which was higher than 77.78% of the control group (P<0.05). After treatment, the PANSS scores of the observation group were significantly lower than those of the control group (P<0.05). The total incidence of adverse reactions in the observation group and control group were 9.30% and 6.98%, respectively, and there was no significant difference between the two groups (P>0.05).

**Conclusion:**

Risperidone combined with olanzapine in the treatment of schizophrenia can effectively reduce symptoms of patients and induce fewer adverse reactions, showing high safety and significant treatment effect.

## INTRODUCTION

Schizophrenia is a kind of mental illness with high incidence rate, but its etiology has not been clear; it is related to family history, psychology, environment and disease.^1^,^2^ Schizophrenic patients have many disorders, for example, thinking and emotion are uncoordinated with mental activities. If no reasonable intervention measures are taken, schizophrenia will damage the cognition of patients, weaken their cognitive function, and increase the family burden.^3^,^4^ In recent years, the treatment of schizophrenia has gradually become the focus of clinical research.^5^

At the current stage, the specific pathogenesis of schizophrenia has not been fully understood, but there are many hypotheses, among which the mental development disorder hypothesis is the most common. Clinical treatment of schizophrenia mainly takes drug treatment preference as the treatment principle to reduce the impact on the vision, hearing and smell of patients. However, there are many kinds of drugs in clinical application, and their effects are different; therefore there is no unified medication standard.^6^ The author found through referring to some references that drugs such as risperidone and olanzapine are often used in the treatment of schizophrenia to relieve the symptoms,^7^,^8^ improve the quality of life, and reduce the pain. In view of the therapeutic effect of the two drugs, our hospital proposed to combine them in the treatment of schizophrenia to observe whether it can enhance the treatment effect.

## METHODS

Ninety-eight schizophrenia patients who were admitted and treated between June 2018 and December 2019 were selected and randomly divided into an observation group and a control group, 49 each group. In the observation group, there were 35 males and 14 females, with an average age of (41.07±4.42) years (26 to 79 years) and with an average disease course of (5.12±0.82) years (2~11 years). In the control group, there were 33 males and 16 females, with an average age of (41.05±4.32) years (2 to 10 years) and an average disease course of (5.76±0.43) years. The general data were homogeneous (P>0.05).

### Inclusion criteria:

1. Being diagnosed as schizophrenia according to the diagnostic basis which was in accordance with the relevant criteria of schizophrenia in the third edition of Chinese Classification and Diagnostic Criteria for Mental Disorders.
2. Being informed with the research content and having signed consent.
3. Not taking drugs used in this study in recent one month.

### Exclusion criteria:

1. Having tumor.
2. Having severe organic diseases.
3. Having AIDS or syphilis.
4. Participating in other clinical researches.
5. Having low compliance.

The control group was treated with risperidone tablets. The initial dose of risperidone tablets (specification: two mg/ten tablets/two plates/one box) was one mg each time, once a day. The dose was gradually increased to 4 mg/day after one week of administration and six mg/day in the second week. Then the dosage was maintained unchanged, usually six mg/day. The treatment lasted for two months.

The observation group was treated with risperidone tablets combined with olanzapine tablets. The control group was treated with risperidone tablets. The usage and dosage of risperidone were the same as those of the control group. The initial dose of olanzapine tablets (specification: seven tablets/box) was 10 mg/day. One week later, the dose was adjusted to five mg/day or 20 mg/day according to the actual condition of patients. The treatment lasted for two months.

Two groups of patients during the medication had high-quality care. The first aspect was daily high-quality care, i.e., intervention on daily life, work and rest and communication. Measures such as adjusting the temperature and humidity, increasing green plants and changing the distribution of environment were taken to make patients feel at home in the ward and promote the recovery of mental state. The patients were guided to work and rest regularly, and the nutrition intake of patients was interfered to maintain nutrition balance. The second aspect was high-quality psychological nursing. Regular psychological evaluation was carried out on patients. Negative psychological emotions of patients were interfered to relieve anxiety and depression and make them maintain a good state of mind. The third aspect was high-quality behavioral nursing. Behavioral intervention was given for patients with severe disease condition. The activity area of patients was limited. Moreover, interesting activities such as chess activities, book corner, etc. were carried out to enrich their daily life.

### Observation index:

The treatment effect was evaluated as significantly effective, effective and ineffective. The total effective rate of the two groups was calculated, and the total effective rate = [(number of significantly effective cases + number of effective cases)/ total number of cases] × 100%. The treatment effect was evaluated as significantly effective if the Positive and Negative Syndrome Scale (PANSS) score significantly decreased and the symptoms significantly relieved. The treatment effect was evaluated as effective if the PANSS score reduced 50% compared to before treatment and the symptoms relieved. The treatment effect was evaluated as ineffective if the PANSS score had no changes and the symptoms had no changes.^9^

The PANSS score of the two groups was observed: PANSS had seven levels of score, and the higher the score was, the more severe the symptoms of patients were.

The cognitive functions of the two groups were observed: the evaluation was performed using Wisconsin Card Sorting Test (WCST), and 128 cards were classified according to the color, shape and quantity; the computer automatically judged whether the choice was right or wrong, and finally the conclusion report was obtained.^10^

The occurrence of adverse reactions in the two groups was observed: the adverse reactions mainly included headache, insomnia, dry mouth, constipation, etc., and the total incidence of adverse reactions was calculated.

### Statistical analysis:

The data were processed by SPSS 25.0. The count data was expressed as rate (%), and the comparison between groups was performed by X^2^ test. The measurement data was expressed by Mean±SD, and the comparison between groups was performed with t test. P<0.05 meant the difference had statistical significance.

## RESULTS

The total effective rate of the observation group was 95.35 %, higher than 79.17 % in the control group (P<0.05; Table-I). Before treatment, there was no significant difference in the PANSS scores between the two groups (P<0.05); after treatment, the PANSS scores of the observation group were lower than those of the control group (P<0.05; Table-II).

**Table-I:** Treatment effect between two groups (%).

Group	Observation group	Control group	t	P
Significantly effective	40	32		
Effective	7	7		
Ineffective	2	10		
Total effective rate	45 (95.92)	39 (79.59)	5.174	<0.05

**Table-II:** PANSS scores between the two groups before and after treatment.

Group		Observation group	Control group	t	P
Positive symptoms	Before treatment	26.35±3.37	26.36±3.39	0.013	>0.05
	After treatment	13.65±1.10	16.75±2.08	7.158	<0.05
Negative symptoms	Before treatment	24.07±0.08	24.08±0.05	0.652	>0.05
	After treatment	11.09±0.55	15.86±1.08	14.279	<0.05

Before treatment, there was no significant difference in the classification completion number, random error number, continuous error number and correct times between the two groups (P<0.05); after treatment, the scores of classification completion number and correct times in the observation group were higher than those in the control group, while the scores of random error number and continuous error number in the observation group were lower than those in the control group, and the differences were statistically significant (P<0.05; Table-III).

**Table-III:** WCST results between the two groups.

Group		Observation group	Control group	t	P
Classification completion number	Before treatment	3.05±1.06	3.08±1.03	0.128	>0.05
	After treatment	3.97±0.22	3.30±0.57	11.187	<0.05
Correct times	Before treatment	25.73±3.11	25.78±3.08	1.263	>0.05
	After treatment	29.97±1.86	26.11±2.85	9.368	<0.05
Random error number	Before treatment	19.66±9.14	19.67±9.13	1.273	>0.05
	After treatment	8.01±2.57	12.58±7.13	11.657	<0.05
Continuous error number	Before treatment	20.46±9.98	20.73±9.17	0.637	>0.05
	After treatment	13.01±8.15	18.98±12.66	13.198	<0.05

There was one case of headache, one case of insomnia, one case of xerostomia and one case of constipation in the observation group, and the total incidence rate was 8.16%. In the control group, there was one case of headache, zero case of insomnia, one case of xerostomia and one case of constipation, and the total incidence rate was 6.12%. There was no significant difference in the total incidence of adverse reactions between the two groups (X^2^=0.157, P>0.05).

## DISCUSSION

Schizophrenia is the result of a variety of factors. Although the specific pathogenesis of schizophrenia has not been fully defined clinically, it is considered that it is closely related to the individual psychological susceptibility quality and external environment.^11^ The clinical symptoms of schizophrenia are complex, involving cognitive function, volitional behavior, emotion, thinking, perception, etc., and individual differences are large.^12^ At present, there are more than 160 kinds of antipsychotic drugs in clinic, but most of them play their roles through the mechanism of medium blocking, which will affect the thalamic pituitary gonadal axis to a certain extent.^13^,^14^

Both olanzapine and risperidone are commonly used antipsychotics in clinic, but their mechanism of action, efficacy and adverse reactions are different. Risperidone can effectively combine with dopamine D2 receptor and 5-hydroxytryptamine receptor and has good effect in improving positive symptoms such as behavior disorder and emotional disorder and negative symptoms such as loss of willpower. However, it should be noted that risperidone has no significant effect on cognitive dysfunction of patients, and the cognitive ability of patients still needs to be improved.^15^ Olanzapine is a new type of antipsychotic drug with high affinity, which can bind with multiple receptors such as 5-hydroxytryptamine, dopamine D, α - epinephrine and histamine H. The biggest difference between olanzapine and risperidone is that olanzapine can prevent the discharge of dopaminergic neurons (midbrain limbic system), protect the striatum motor function of patients, and improve the cognitive impairment of patients.^16^ From the perspective of pharmacological effect, olanzapine combined with risperidone may produce some synergistic effects similar to that of the second generation antipsychotics combined with the first generation drugs.

In this study, the total effective rate of the observation group was significantly higher than that of the control group; and compared with the control group, the PANSS score of the observation group was lower after treatment, which was basically similar to the research results of Li.^17^ The above result showed that the effect of risperidone combined with olanzapine was more ideal compared with risperidone alone in the treatment of schizophrenia. In addition, the scores of the classification completion number and correct times in the observation group were higher than those in the control group, while the scores of random errors and continuous errors in the observation group were lower than those in the control group, which further verified that olanzapine combined with risperidone was effective in the treatment of schizophrenia and it could effectively improve the cognitive function of patients.

Generally, the combined drug treatment will increase the occurrence of adverse reactions, but the occurrence observation of adverse reactions in this study showed that there was no significant difference in the adverse reactions between the combined drug treatment and single drug treatment (P > 0.05), indicating that the safety of the combined drug treatment.

## CONCLUSION

Olanzapine combined with risperidone has significant clinical effect in the treatment of schizophrenia and is conducive to improve the cognitive function of patients. However, due to the limited follow-up time, this study did not carry out a comparative study on the improvement of the long-term quality of life of the two groups of patients, which needs further subsequent study.

### Authors’ Contribution:

**LJY & XQ:** Study design, data collection and analysis.

**LJY & XQ:** Manuscript preparation, drafting and revising.

**LJY:** Review, final approval of manuscript and is responsible for integrity of the study.

## References

[1] Abraham KR, Kulhara P (2018). The efficacy of electroconvulsive therapy in the treatment of schizophrenia. A comparative study. Br J Psychiat.

[2] Kambeitz J, Abi-Dargham A, Kapur S, Howes OD (2018). Alterations in cortical and extrastriatal subcortical dopamine function in schizophrenia:systematic review and meta-analysis of imaging studies. Br J Psychiat J Mental Sci.

[3] Zhang S (2018). Efficacy of amisulpride combined with clozapine in the treatment of refractory schizophrenia. Chin J Mod Drug Appl.

[4] Myhrman A, Rantakallio P, Isohanni M, Jones P, Partanen U (2018). Unwantedness of a pregnancy and schizophrenia in the child. Br J Psychiat.

[5] Siris SG, Sellew AP, Frechen K, Cooper TB, Mandell J, Casey E (1988). Antidepressants in the treatment of post-psychotic depression in schizophrenia:drug interactions and other considerations. Clin Chem.

[6] Lieberman JA (2018). Disease modifying effects of antipsychotic drugs in schizophrenia:a clinical and neurobiological perspective. World Psychiat Offic J World Psychiat Assoc.

[7] Kong LG, Lin CG, Huang QQ, Li YB, Xu DL, Xie HL (2018). Influence of 5 antipsychotic drugs on liver function and heart function in the treatment of schizophrenia. Lab Med.

[8] Wang Y, Chen ZJ, Yang Y (2017). Effect and safety of olanzapine in the treatment of elderly patients with schizophrenia. China J Health Psychol.

[9] Zhang Y, Kang CY, Yuan J, Zeng L, Wei YJ, Xu L, Yang JZ (2017). The association of catechol-O-methyl transferase gene polymorphism with the clinical efficacy of risperidone in the treatment and cognitive function in patients with schizophrenia. Chin J Behav Med Sci.

[10] Zhang SX, Shi JY, Ye FH, Zheng GD, Wang J, Wu KW, Dai T (2016). A controlled study of piperapirone and risperidone in improving symptoms and cognitive function in schizophrenia. Chin Remed Clin.

[11] Ursini G, Punzi G, Chen Q (2018). Convergence of placenta biology and genetic risk for schizophrenia. Nat Med.

[12] Schottle D, Schimmelmann BG, Karow A, et al. (2018). Effectiveness of integrated care including therapeutic assertive community treatment in severe schizophrenia-spectrum and bipolar I disorders:Four-year follow-up of the ACCESS II study. PLoS ONE.

[13] Mellin JM, Alagapan S, Lustenberger C, Lugo CE, Alexander M, Gilmore JH, Jarskog LF, Frohlich F (2018). Randomized trial of transcranial alternating current stimulation for treatment of auditory hallucinations in schizophrenia. Eur Psychiatry.

[14] Jiang H (2018). Analysis of the effects of olanzapine and risperidone on cognitive function in patients with first episode schizophrenia. Guide China Med.

[15] Maneeton N, Maneeton B, Puthisri S, Woottiluk P, Narkpongphun A, Srisurapanont M (2018). Risperidone for children and adolescents with autism spectrum disorder:a systematic review. Neuropsych Dis Treat.

[16] Katagiri H, Taketsuna M, Kondo S, Kajimoto K, Aoi E, Tanji Y (2018). Effectiveness and safety of oral olanzapine treatment transitioned from rapid-acting intramuscular olanzapine for agitation associated with schizophrenia. Neuropsych Dis Treat.

[17] Li ZM (2015). Clinical study of olanzapine combined with risperidone in the treatment of schizophrenia. Mod J Integr Tradit Chin.

